# Selective Forms of Therapy in the Treatment of Inflammatory Bowel Diseases

**DOI:** 10.3390/jcm11040994

**Published:** 2022-02-14

**Authors:** Anna Kofla-Dłubacz, Katarzyna Akutko, Elżbieta Krzesiek, Tatiana Jamer, Joanna Braksator, Paula Grębska, Tomasz Pytrus, Andrzej Stawarski

**Affiliations:** 2nd Department of Paediatrics, Gastroenterology and Nutrition, Faculty of Medicine, Wroclaw Medical University, M. Sklodowskiej-Curie Str. 50/52, 50-367 Wroclawl, Poland

**Keywords:** IBD, pro-inflammatory cytokines, lymphocyte migration, treatment

## Abstract

Selective interference with the functioning of the immune system consisting of the selective blockade of pro-inflammatory factors is a modern, promising, and developing strategy for the treatment of diseases resulting from dysregulation of the immune system, including inflammatory bowel disease. Inhibition of the TNF alpha pathway, group 12/23 cytokines, and lymphocyte migration is used in the treatment of severe or moderate ulcerative colitis and Crohn’s disease. Intracellular signal transduction by influencing the phosphorylation of SAT (signal transducer and activator of transcription) proteins remains in clinical trials.

## 1. Introduction

Inflammatory bowel diseases (IBD), which include two main types: Crohn’s disease (CD) and ulcerative colitis (UC), are chronic diseases of the gastrointestinal tract whose etiology has not been fully elucidated. It is known that the underlying cause of the development of IBD is over-stimulation of pro-inflammatory signaling pathways, falling out of regulatory mechanisms. Increasingly, knowledge of these pathomechanisms is being used in the development of treatment strategies, highly specific for triggers of immune response activation. The inhibition of the accumulation of immune cells, where inflammation develops, and the inhibition of the activity of pro-inflammatory cytokines have become the main goals of the development of new and more effective forms of therapy. In the mucosa of the gastrointestinal tract of people with CD, dysregulation of the functional elements of the immune system is observed, including accumulation and hyperactivity of Th 1 and Th 17 lymphocytes, depending on the excessive production of cytokines, mainly tumor necrosis factor alpha (TNF-α), interleukin (IL)-12, and IL-23. This stimulation results, among others, from the immune system’s response upon stimulation with bacterial antigens in the intestinal lumen. In addition to changing the composition of the intestinal microbiome, the initial impact on the development of the disease may include reduced mucus secretion (the Muc2 phenotype in a mouse model) or a change in the expression of molecules mediating the adhesion and interaction of bacteria with the immune system (variant FUT2). The balance of pro-inflammatory and anti-inflammatory cytokines activity determines the proper functioning of the human immune system, while excessive or insufficient activation of triggering factors is detected in many autoimmune diseases, as well as in neoplastic transformation [1]. Knowledge of the signaling pathways of the stimulation of the inflammatory process allowed for the development of biological drugs is used in the therapy of IBD. TNF-α inhibitors (infliximab-IFX and adalimumab-ADA) were the first monoclonal antibodies used in the treatment of patients with IBD.

## 2. Tumor Necrosis Factor Alpha

TNF-α is a central pro-inflammatory cytokine produced mainly in macrophages and monocytes. TNF-α has a pleiotropic effect on cells, and among others, it stimulates the migration of NF kB from intracellular plasma to the nucleus. It stimulates the production of various pro-inflammatory molecules, as well as cell proliferation, differentiation, and angiogenesis, and has a pro-thrombotic effect. Its action ultimately leads to cell necrosis or apoptosis. TNF-α acts on cells by binding to cell membrane receptors 55 kDa TNFR-1 or 75 kDa TNFR-2. TNF-α plays a crucial role in both the formation and maintenance of inflammation in many tissues and organs, including the gut. Higher serum levels of TNF-α than in the healthy population are observed in both UC and CD patients. In addition, there is an increased number of cells secreting TNF-α in the inflamed intestinal mucosa in the course of IBD. For this reason, in recent decades, research has been carried out on substances that block the action of TNF-α, including precisely in the treatment of IBD [2,3].

The first drugs tested were TNF-α inhibitors. This group includes monoclonal antibodies, such as IFX and ADA, as well as antibody fragments, such as certolizumab (CER) and the fusion proteins etanercept (ETA). The mechanism of their action is not fully understood, and due to the complexity of TNF-α signaling, it is likely that the interaction of drugs is not only simple blockades [4].

IFX is a chimeric human-mouse monoclonal antibody (IgG1) directed against TNF-α with a molecular weight of 149 kDa. The human component constitutes 75% of it. IFX inhibits the activity of TNF-α. It reduces the infiltration of inflammatory cells into the tissue, as well as the expression of cell adhesion molecules, chemotactic activity and tissue degradation. It contributes to the death of activated lymphocytes and monocytes.

The recommended dose of IFX is 5 mg/kg body weight given as an intravenous infusion. The induction of remission is three doses at intervals of 0–2–6 weeks, and to maintain remission, administration is necessary at intervals of 8 weeks [5].

ADA is a recombinant pure human monoclonal antibody (IgG1) with a size of 148 kDa. ADA binds specifically to human TNF-α, which prevents this cytokine from binding to the p55 and p75 receptors on the surface of cells. Thus, ADA inhibits its pro-inflammatory activity. By changing the activity of TNF-α, ADA also indirectly influences the concentration of cellular adhesion particles that directly regulate the leukocyte migration process. The drug is administered subcutaneously. In the induction phase of remission, the ADA dose is 160/80 mg–80/40 mg (0–2 weeks), followed by 40 mg every 2 weeks for the next 12 weeks. In the maintenance phase of remission, 40 mg should be administered every 2 weeks [6].

Many clinical trials, including ACCENT I (A Crohn’s Disease Clinical Trial Evaluating Infliximab In a New Long-term Treatment Regimen), have assessed the safety and efficacy of anti-TNF-alpha antibodies in the treatment of IBD. The study showed that at 52 weeks of treatment, steroid-free remission was observed more frequently in patients receiving IFX than in placebo, and this difference was statistically significant (24% vs. 9%; *p* = 0.031). Similarly, the CHARM (Crohn’s Trial of The Fully Human Antibody Adalimumab for Remission Maintenance) study confirmed that ADA treatment is also effective in the treatment of CD; after 56 weeks of treatment, remission without glucocorticoids was observed in 29% of patients with ADA, with only 6% receiving a placebo (*p* < 0.001). The benefits of using anti-TNF antibodies have also been demonstrated by other clinical trials: CLASSIC, EXTEND, ULTRA [7].

IFX was registered in CD in adults in 1998, in children in 2006, in UC in adults in 2005, and in children in 2011. ADA was registered in the treatment of CD in adults in 2007, in children in 2012, and also in 2012 in the treatment of adults with UC [8], and in children in 2021 [9]. IFX and ADA are the longest-used biologics in the treatment of IBD and remain the only biologics approved for the treatment of children.

## 3. The Group of Interleukin-12 Cytokines

The group consisted of interleukins (IL) 12, 23, 27 35. IL-12, IL-23, and IL-27 are secreted by previously activated antigen-presenting cells (APC), mainly macrophages and dendritic cells. IL-35 is secreted by lymphocytes, both regulatory T (T reg) and B-type lymphocytes. IL-12 and IL-23 have a pro-inflammatory effect, as they activate NK cells (natural killer) and stimulate the process of CD4 + cell differentiation into Th 1 and Th 17 [10]; IL-27 and 35 have an immunosuppressive effect [10,11].

A special feature of the IL-12 group cytokines is the heterodimeric structure of the α (p19, -28, p35) and β (p40, Ebi3) component units. Il-12 contains the p40-p35 subunits, while IL 23 has p40-p19. The stimulation of the pro-inflammatory intracellular signaling pathway is carried out by the connection of the p40 protein subunit with the membrane receptor IL-12Rβ1 of effector cells. The selective blockade of the IL-12 group of cytokines has been shown to be effective in inhibiting an excessive inflammatory response in the gastrointestinal mucosa. Ustekinumab and briakinumab are drugs that abolish the functional effect of IL-12 and IL-23 activity, while risankizumab, mirikizumab and brazicumab inhibit the IL-23 stimulation pathway [12].

Ustekinumab, as a monoclonal antibody (IgG1κ) that specifically binds to the p40 subunit, prevents an interaction with the receptor, and subsequently, the activation of the immune system-dependent IL-12/23. In a clinical trial in adults who did not respond to treatment with TNF-antagonists or experienced significant adverse events during treatment, which assessed the efficacy of induction treatment with intravenous ustekinumab, it was shown that in patients treated with ustekinumab, the clinical response was statistically significantly more frequent compared to those receiving placebo (34.3% vs. 21.5%; *p* < 0.001), (UNITI-1). Additionally, 33.7% of patients who did not benefit from standard therapy or who discontinued therapy due to adverse events had a significant clinical response (*p* < 0.001) (UNITI-2). During the maintenance phase, remission at week 44 of the study was maintained in 53.1% of patients receiving the drug by subcutaneous injection at 8 week intervals, and in 48.8% of patients who received the drug every 12 weeks, though only in 35.9% of subjects receiving the placebo (*p* = 0.005; *p* = 0.04), (IM-UNITI) [13]. The indications for the use of ustekinumab are CD and UC in people who have not responded to conventional treatment and anti-TNF-α therapy, or who have developed side-effects that make it impossible to continue therapy [14]. In the pediatric population, the drug has not been approved yet, and the necessary clinical trials are underway. However, therapy is possible for patients who have exhausted the existing conventional methods of treatment, each time after obtaining the opinion of the ethics committee [15]. Among the indications other than IBD, the drug is used in plaque psoriasis in adults and children and adolescents [14]. In IBD, the drug is administered according to the following schedule: a single induction dose administered intravenously, and subsequent doses at 8 week intervals administered subcutaneously [14].

## 4. Leukocyte Migration as an Expression of the Immune System’s Hyperreactivity

Leukocyte migration to the inflamed tissue is dependent on the expression of leukocyte membrane proteins (integrins) that interact with cell adhesion molecules (CAM), promoting the migration of lymphocytes into tissues. The process of releasing immune system cells from the lymph nodes into the lymphatic vessels is related to the interaction of S1P receptors (sphingosine-1-phosphate receptors) present on the surface of lymphocytes with their ligands. Th lymphocytes, which exercise immunological supervision in the gastrointestinal tract, express integrin α4β7, which has an affinity for the mucosal addressin cell adhesion molecule-1 (MAdCAM-1) of intestinal vascular endothelial cells and is responsible for the activation and maintenance of the inflammatory process-lymphocytes accumulation in the intestine. This mechanism is one of the pathways of an excessive inflammatory response in IBD, including CD. Thus, inhibition of the interaction between the MAdCAM-1 integrin may have important therapeutic significance. Drugs that inhibit Th (leukocyte migration) migration to the gastrointestinal tract by blocking specific leukocyte integrins are vedolizumab (anti-α4β7), abrilumab (anti-α4β7 IgG2), etrolizumab (anti-β7), α4-specific small molecule AJM300 (orally active small molecule inhibitor of α4) and PN-943 (oral gastrointestinal-restricted peptide antagonist of α4β7). Blockade of the MAdCAM-1 molecule is another strategy leading to the inhibition of the interactions: integrin alpha 4, beta 7-MAdCAM-1, and an antibody with the potential to act in this way is PF-00547659 [16]. Of these drugs, vedolizumab is approved for the treatment of UC and CD in adults. The remaining drugs mentioned have potential value in the effective treatment of IBD [8].

Vedolizumab is an anti-α4β7 integrin humanized IgG1 monoclonal antibody. Natalizumab, an anti-α4 integrin antibody, was the prototype of therapy targeting the interaction of lymphocytic integrins with adhesive molecules due to significant side effects, including progressive multifocal leukoencephalopathy, is not currently used. Evaluation of the efficacy and safety of vedolizumab in the treatment of IBD, both in the induction phase and the maintenance phase of remission, was the main goal of the GEMINI studies (GEMINI 1-UC; GEMINI 2-CD, GEMINI distant safety). The studies showed that the effectiveness of vedolizumab therapy was statistically higher than in the placebo-controlled groups, both in the induction phase and in the maintenance of remission [17,18]. Vedolizumab is administered intravenously in the induction phase at weeks 0, 2 and 6, then during the maintenance phase, also by intravenous infusions at 8 week intervals, or every 4 weeks if there is a decrease in response [19].

Etrolizumab is a monoclonal antibody directed selectively against the β7 subunit of the α4β7 and αEβ7 integrins. The phase II study showed that drug-treated patients with moderate to severe UC were more likely to achieve clinical remission than the placebo at week 10 [20]. The results of phase III trials to date do not provide a clear answer as to whether etrolismumab is more effective than the placebo or TNF-α antagonists, especially in the maintenance phase of remission in UC patients. Further studies are needed to finally determine the efficacy of etrolizumab in the treatment of IBD [21]. Importantly, no serious adverse events were reported with etrolizumab therapy [20,21].

## 5. Sphingosine-1-Phosphate Receptor Modulators

Another drug limiting lymphocytic migration is the orally bioavailable ozanimod. It is a selective modulator of sphingosine-1-phosphate receptors. There are five subtypes of S1P receptors: S1P1-5R. They are found on many cells of the body, but the S1P1 and S1P5 isoforms are present mainly in immune system cells and they are the center of action of ozanimod, which limits the effect of the drug on other organs [22]. According to the Food and Drug Administration (FDA) and *European Medicines Agency* (EMA), treatment with ozanimod is currently approved in adult patients with relapsing-remitting MS [23]. The TOUCHSTON, a phase 2 placebo-controlled trial and the True North, a phase 3 placebo-controlled trial, and Phase II and III studies have shown efficacy in remission induction and maintenance therapy in adults with moderate to severe UC. At the end of the induction and maintenance treatment period, the proportion of patients who achieved clinical improvement, a change in Mayo score, and mucosal healing treated with ozanimod was greater than with the placebo. However, the adverse event profile was comparable between the ozanimod and placebo groups [24]. Based on the results of these trials in 2021, the FDA approved ozanimod for adults with moderately to severely active UC [25]. The STEPSTONE, a phase 2 study, was being conducted in adult patients with moderate and severe CD. Clinical response and clinical remission were seen in 56.5% and 39.1% of subjects at week 12, respectively. Ozanimod is well-tolerated in patients with CD and is consistent with that observed in other patient populations (UC and SM). No serious side effects have been reported (23). Four phase 3 clinical trials with ozanimod are currently ongoing in adult patients with CD (NCT03467958, NCT03440385, NCT03440372, and NCT03464097) [26].

A drug with a similar method of action is etrasimod. Previous studies show that etrasimod is a safe drug and leads to significant clinical and endoscopic improvement in patients with moderate or severe UC. In 2020, the results of a randomized phase 2 study in which patients with UC were qualified, were presented in the Journal of Gastroenterology. The multicentre international study, which lasted 12 weeks, enrolled 156 subjects who were assigned to three groups: a 1 mg or 2 mg study drug (*n* = 52, *n* = 50) and placebo (*n* = 54). The primary endpoint was the mean improvement of the Mayo Clinical Score from baseline to week 12. Etrasimod 2 mg led to a statistically significant improvement in the Mayo Clinical Score compared to pre-trial scores than placebo (0.99 points different from the placebo; *p* = 0.009); the 1 mg dose also improved the baseline value (0.43 points more than placebo), but in this case, the effect was not statistically significant (*p* = 0.15). In patients receiving the drug in a 2 mg dose, regression of endoscopic changes was also statistically significantly more often than in patients receiving a placebo (41.8% vs. 17.8%, *p* = 0.003). Etrasimod therefore appears to have a good safety profile. Most of the adverse effects were mild or moderate in severity [27]. The study showed that in patients with moderate to severe UC, 2 mg of etrasimod led to significant clinical and endoscopic improvement. Following completion of the OASIS study, patients had the option of continuing etrasimod 2 mg treatment for an additional 34–40 weeks as part of an open-label extension (OLE protocol) [28]. The study was conducted in 14 countries and 51 clinical sites. A total of 118 patients were enrolled in the OLE protocol, 112 of whom received 2 mg of etrasimod. The study was completed by 92/112 (82%) patients treated with etrasimod 2 mg according to the OLE protocol. Although in this group, the drug-related adverse events were observed in 67/112 (60%) patients, they were only mild or moderate (94%). The most common symptoms were worsening of the underlying disease and anemia. 64% of patients achieved a clinical response, 33% achieved clinical remission, and 43% achieved endoscopic improvement. Clinical response, clinical remission or endoscopic improvement at week 12 was kept at the end of the study in 85%, 60%, and 69% of the study group, respectively. A total of 22% of patients maintained steroid-free clinical remission. Thus, long-term use of etrasimod at a dose of 2 mg per day was safe and brought measurable benefits to the patients [28].

## 6. Janus Kinases Inhibitors

Therapies with the use of monoclonal antibodies used in the treatment of autoimmune diseases have fundamentally changed the patient prognosis, improved their quality of life, and thus also reduced the social effects resulting from the chronicity of the disease. However, other new therapeutic strategies are also under development, using particles modifying the transduction of intracellular signals along the cytokine receptor pathway and growth factors in the cell membrane to the cell nucleus, by influencing the signal transducer and activator of transcription Janus kinases (JAK, Janus-activated kinases). Tofacitinib is a drug that blocks the activity of three types of JAK. It is an oral drug used to treat rheumatoid arthritis and psoriatic arthritis, as well as for UC. Other medicines that belong to the class of JAK inhibitors are filgotinib and upadacitinib [29]. In vitro studies in human T cells showed that tofacitinib blocks IL-6-, IFN-γ-, and IL-12-dependent signaling from JAK3 receptors, and also decreased signaling from JAK1 and JAK2 receptors. As a result, the secretion of pro-inflammatory cytokines and mediators related to the immune reaction is limited. Additionally, tofacitinib prevents the differentiation of CD4 + T cells into Th1, Th2, and Th17 lymphocytes in mice. In addition, tofacitinib modulates the immune response by altering lipopolysaccharide signaling. In summary, tofacitinib significantly suppresses the immune response, which plays an important role in the etiopathogenesis of IBD [30,31].

The efficacy of tofacinitib in adult patients with moderate or severe UC has been studied in several clinical trials (phase 3). The efficacy of induction therapy was assessed in OCTAVE Induction 1 and 2—multicentre, randomized, double-blind, placebo-controlled trials. A total of 139 patients (OCTAVE 1–598 and OCVTACE 2–541 patients) were assigned to receive induction treatment with tofacitinib or a placebo for 8 weeks. In OCTAVE 1, there were statistically significantly more patients who achieved clinical remission in the tofacinitib group than in the placebo group (18.5% vs. 8.2%, *p* < 0.01). Efficacy in induction remission therapy was reported more frequently in patients receiving tocafinibib than in patients receiving the placebo (31.3% vs. 15.6%, *p* < 0.001). The effectiveness of tofacinitib was comparable in patients who had previously been treated with TNF-α inhibitors and those who had not been treated. The results of the OCTAVE 2 study were similar to the results from OCTAVE 1 [27,31,32]. The OCTAVE study also assessed the efficacy of tofacitinib in the maintenance treatment of UC. It was shown that at 52 weeks of treatment, remission was observed more frequently in patients receiving tofacitinib compared to the placebo group (tofacitinib dose: 5 mg—34.3%, 10 mg—40.6% vs. 11.1%), and from week 4 of the study there was a significant difference between patients on the placebo compared to those receiving tofacitinib. Among patients who responded well to maintenance therapy, those receiving tofacitinib were more likely to maintain glucocorticoid-free remission at 24 and 52 weeks than those receiving the placebo (*p* < 0.001) [27,32]. A systematic review by Pantavou et al. confirmed the efficacy and safety of tofacinitib in the treatment of UC. It was also noted that tofacitinib appeared to be more effective than adalimumab and golimumab in maintaining remission and in the improvement of endoscopic changes in adult patients with UC [33]. In 2018, based on the results of the quoted studies, the FDA approved tofacitinib for the treatment of moderate to severe UC in adult patients who did not respond adequately to conventional therapy [34]. Phase III clinical studies are ongoing to evaluate the efficacy, safety and pharmacokinetics of tofacitinib in children with moderately or severely active UC [35]. Phase II, randomized, blinded, and placebo-controlled multicentre studies of the efficacy of tofacinitib have also been conducted in adult patients with moderate to severe CD. However, the efficacy of tofacinitib in inducing and/or maintaining remission has not been demonstrated to be statistically significantly higher than the placebo [36].

There are also ongoing clinical trials with other JAK inhibitors in the treatment of IBD, both in CD and UC, including phase 2 trials of upadacitinib and filgotinib [37]. Upadacitinib is a selective JAK1 inhibitor. In the CELEST trial in patients with moderate to severe CD, the efficacy of Upadacitinib was greater than the placebo in inducing clinical and endoscopic remission (*p* < 0.01). However, it is noteworthy that the achievement of clinical remission was dose-dependent. Similar results were obtained in the U-ACHIEVE study evaluating the efficacy of Upadacitinib in patients with moderately or severely active UC compared to the placebo in induction of clinical remission (*p* = 0.002 for the 45 mg dose) and endoscopic remission (*p* < 0.05 regardless of dose). However, there is a need for further, more detailed studies on a large population of patients to confirm these observations [38]. Another selective JAK1 inhibitor is filgotinib. The efficacy of orally administered filgotinib in inducing and maintaining remission in adult UC patients was assessed in the SELECTIVE study, and it was shown that treatment with 200 mg oral filgotinib was associated with significantly more clinical remission at 10 and 58 weeks in filgotinib-treated patients than in placebo-treated patients (*p* = 0.003; *p* < 0.0001) [39]. Filgotinib obtained a positive opinion from the EMA and was approved for the treatment of adult UC patients in the European Union in November 2021 [40]. It was shown that patients receiving oral filgotinib at a dose of 200 mg achieved clinical remission significantly more often than those receiving the placebo (*p* = 0.0077). However, there was no statistically significant difference between patients receiving the drug and those taking a placebo in the induction of endoscopic remission, mucosal healing, or deep remission (*p* = 0.31; *p* = 0.82; *p* = 0.31). A phase III study is currently being conducted to assess the effect of filgotinib on the course of CD in adults [41]. Another moderately selective JAK 3 inhibitor evaluated in Phase II clinical trials for the treatment of adult UC patients was peficitinib administered orally at various doses. Higher clinical and endoscopic remission rates and mucosal healing rates were observed in patients receiving higher doses of peficitinib compared to those receiving a placebo, but these differences were not statistically significant. However, it is noteworthy that side-effects were observed more frequently in patients receiving peficitinib than in patients receiving the placebo [42].

TYK2 belongs to the JAK-STAT family of proteins, which are an important element of intracellular signaling stimulated by various cytokines. The use of the TYK2/JAK1 inhibitor brepocitinib has been reported to be effective in the treatment of plaque psoriasis. Phase 2 trials are also ongoing in combination with brepocitinib and a selective JAK3 inhibitor known as PF-06651600 in patients with both moderate to severe UC and CD [43].

## 7. Interleukin-6 Inhibitors

Interleukin-6 (IL-6) is known to be a multidirectional cytokine. It stimulates, among others, the migration of phagocytic cells and lymphocytes to the place where chronic inflammation takes place. Thus, IL-6 can have a significant influence on the development and maintenance of IBD. It has also been shown that the concentration of IL-6 is often higher in serum and in the inflamed intestinal wall in patients with severe CD [44].

PF-04236921 is a human monoclonal antibody against IL-6. The ANDANTE I and II clinical trials assessed the efficacy and safety of PF-04236921 in the treatment of adults with moderate to severe CD who had not benefited from treatment with anti-TNF alpha agents. Various subcutaneous doses of drugs (10 mg, 50 mg, and 200 mg) have been studied, and it has been shown that only patients receiving 50 mg of the drug achieved a significantly better clinical response at week 12 than the placebo group (47.4% vs. 28%, *p* = 0.004). It is also important that serious adverse events (gastrointestinal perforation and suppuration) have been reported during, and even after treatment completion. Therefore, the safety assessment of PF-04236921 treatment will be extremely important in future clinical trials [43,45].

Attention is also drawn to the fact that the pro-inflammatory action of IL-6 is the result of transmembrane signaling resulting from stimulation of the soluble membrane receptor in the presence of the gp130 co-receptor. It has been shown in preclinical studies that blocking signal transduction by a special decoy protein sgp130Fc (olamkicept) can inhibit pro-inflammatory processes without blocking the IL-6 receptor itself. This avoids immunosuppression, and therefore has significant benefits. In FUTURE Phase II studies, olamkicept was used in 16 patients with IBD. The authors concluded that the drug was well-tolerated, the clinical response was noted in 44% of patients, and clinical remission was noted in 19% of patients. There is a need for further studies to evaluate the safety and efficacy of this new type of immunoregulatory therapy in IBD patients [46].

## 8. IL-22Fc Fusion Protein

There are also studies aimed at finding a way to induce mucosal healing without the need for anti-inflammatory action and inducing immunosuppression in patients. It promotes the secretion of antimicrobial substances; the enhancement of these effects by appropriate stimulation of the IL-22 pathway of action may therefore promote the regeneration of tissues, including the intestinal mucosa.

Based on those observations, a study was carried out on subjects who received IL-22 associated with the crystallizing part of human immunoglobulin G4 (Fc), creating the so-called fusion protein-IL-22Fc. They showed higher concentrations of mediators of the IL-22 pathway, which may also directly affect the tissue regeneration of inflamed lesions in the course of IBD, but without inducing immunosuppression and the resulting consequences for the patient. The above reports must be confirmed in further clinical trials on a large number of patients [43,47,48].

## 9. Phosphodiesterase 4 Inhibitors

Phosphodiesterases (PDE1-PDE11) are enzymes involved in the transformation of intracellular cAMP. Their activity results in the activation of the nuclear transcription factor kappaB (NF-κB), which promotes the development of inflammation (e.g., by stimulating the secretion of TNF-α and inhibiting the secretion of anti-inflammatory cytokines). Thus, inhibition of these enzymes may reduce non-specific inflammation, hence the need to investigate the possibility of using PDE4 inhibitors as a form of IBD treatment [43]. The efficacy and safety of orally administered apremilast, a PDE4 inhibitor, in adult UC patients was assessed in a phase II randomized, double-blind, placebo-controlled study. Clinical remission was observed in patients taking apremilast more than in the placebo group, but a statistically significant difference was only shown in patients receiving 30 mg of the drug compared to the placebo (31.6% vs. 12.1%; *p* = 0.01). The authors emphasize, however, that the use of apremilast contributed to a significant decrease in inflammatory markers (C-reactive protein in the blood and calprotectin in the feces) [48].

## 10. Summary

The increase in the incidence of autoimmune diseases in population requires an emphasis on the search for new therapeutic strategies in the care of patients not only with IBD. Continuation of research on immunological mechanisms in the course of autoimmune diseases and further identification of both pro-inflammatory and anti-inflammatory triggers makes it possible to achieve highly selective forms of therapy with a limited number of side-effects in the future.

## References

[1] Yan J., Smyth M.J., Teng M.W.L. (2018). Interleukin (IL)-12 and IL-23 and Their Conflicting Roles in Cancer. Cold Spring Harb. Persp. Biol..

[2] Idriss H.T., Naismith J.H. (2000). TNF alpha and the TNF receptor superfamily: Structure-function relationship(s). Microsc. Res. Tech..

[3] Tracey D., Klareskog L., Sasso E.H., Salfeld J.G., Tak P.P. (2008). Tumor necrosis factor antagonist mechanisms of action: A comprehensive review. Pharmacol. Ther..

[4] Billmeier U., Dieterich W., Neurath M.F., Atreya R. (2016). Molecular mechanism of action of anti-tumor necrosis factor antibodies in inflammatory bowel diseases. World J. Gastroenterol..

[5] Remicade SoPC. https://ec.europa.eu/health/documents/community-register/2004/200409208252/anx_8252_pl.pdf.

[6] Humira SoPC. https://ec.europa.eu/health/documents/community-register/2007/2007100932109/anx_32109_pl.pdf.

[7] Cholapranee A., Hazlewood G.S., Kaplan G.G., Peyrin-Biroulet L., Ananthakrishnan A.N. (2017). Systematic review with meta-analysis: Comparative efficacy of biologics for induction and maintenance of mucosal healing in Crohn’s disease and ulcerative colitis controlled trials. Aliment. Pharm. Ther..

[8] Breton J., Kastl A., Conrad M.A., Baldassano R.N. (2020). Positioning Biologic Therapies in the Management of Pediatric Inflammatory Bowel Disease. Gastroenterol. Hepatol..

[9] HUMIRA® (adalimumab) Receives FDA Approval to Treat Pediatric Patients Living with Moderately to Severely Active Ulcerative Colitis. https://www.prnewswire.com/news-releases/humira-adalimumab-receives-fda-approval-to-treat-pediatric-patients-living-with-moderately-to-severely-active-ulcerative-colitis-301235101.html.

[10] Vignali D.A., Kuchroo V.K. (2012). IL-12 family cytokines: Immunological playmakers. Nat. Immunol..

[11] Yoshida H., Hunter C.A. (2015). The immunobiology of interleukin-27. Annu. Rev. Immunol..

[12] Kashani A., Schwartz D.A. (2019). The Expanding Role of Anti-IL-12 and/or Anti-IL-23 Antibodies in the Treatment of Inflammatory Bowel Disease. Gastroenterol. Hepatol..

[13] Feagan B.G., Sandborn W.J., Gasink C., Jacobstein D., Lang Y., Friedman J.R., Blank M.A., Johanns J., Gao L.-L., Miao Y. (2016). Ustekinumab as Induction and Maintenance Therapy for Crohn’s Disease. N. Engl. J. Med..

[14] Stelara-Summary of Product Characteristics. https://www.ema.europa.eu/en/documents/product-information/stelara-epar-product-information_en.pdf.

[15] Kapoor A., Crowley E. (2021). Advances in Therapeutic Drug Monitoring in Biologic Therapies for Pediatric Inflammatory Bowel Disease. Front. Pediatrics.

[16] Gubatan J., Keyashian K., Rubin S.J.S., Wang J., Buckman C.A., Sinha S. (2021). Anti-Integrins for the Treatment of Inflammatory Bowel Disease: Current Evidence and Perspectives. Clin. Exp. Gastroenterol..

[17] Lam M.C., Bressler B. (2014). Vedolizumab for ulcerative colitis and Crohn’s disease: Results and implications of GEMINI studies. Immunotherapy.

[18] Vermeire S., Loftus E.V., Colombel J.F., Feagan B.G., Sandborn W.J., Sands B.E., Danese S., D’Haens G.R., Kaser A., Panaccione R. (2017). Long-term Efficacy of Vedolizumab for Crohn’s Disease. J. Crohns Colitis.

[19] Entyvio-Summary of Product Characteristics. https://www.ema.europa.eu/en/documents/product-information/entyvio-epar-product-information_en.pdf.

[20] Cai Z., Wang S., Li J. (2021). Treatment of Inflammatory Bowel Disease: A Comprehensive Review. Front. Med..

[21] Agrawal M., Verstockt B. (2022). Etrolizumab for ulcerative colitis: Beyond what meets the eye. Lancet Gastroenterol. Hepatol..

[22] Verstockt B., Ferrante M., Vermeire S., Van Assche G. (2018). New treatment options for inflammatory bowel diseases. J. Gastroenterol..

[23] Fronza M., Lorefice L., Frau J., Cocco E. (2021). An Overview of the Efficacy and Safety of Ozanimod for the Treatment of Relapsing Multiple Sclerosis. Drug Des. Devel. Ther..

[24] Sandborn W.J., Feagan B.G., D’Haens G., Wolf D.C., Jovanovic I., Hanauer S.B., Ghosh S., Petersen A., Hua S.Y., Lee J.H. (2021). Ozanimod as Induction and Maintenance Therapy for Ulcerative Colitis. N. Engl. J. Med..

[25] Bristol-Myers Squibb Company (2021). Zeposia Prescribing Information. Zeposia U.S. Product Information.

[26] https://clinicaltrials.gov/ct2/resultsterm=ozanimod&cond=Crohn+Diease&draw=2&rank=5#rowId4,%20access%2001.02.2022.

[27] Sandborn W.J., Peyrin-Biroulet L., Zhang J., Chiorean M., Vermeire S., Lee S.D., Kuhbacher T., Yacyshyn B., Cabell C.H., Naik S.U. (2020). Efficacy and Safety of Etrasimod in a Phase 2 Randomized Trial of Patients With Ulcerative Colitis. Gastroenterology.

[28] Vermeire S., Chiorean M., Panés J., Peyrin-Biroulet L., Zhang J., Sands B.E., Lazin K., Klassen P., Naik S.U., Vabell C.H. (2021). Long-term Safety and Efficacy of Etrasimod for Ulcerative Colitis: Results from the Open-label Extension of the OASIS Study. J. Crohns Colitis.

[29] Fernández-Clotet A., Castro-Poceiro J., Panés J. (2019). JAK Inhibition: The Most Promising Agents in the IBD Pipeline?. Curr. Pharm. Des..

[30] Ghoreschi K., Jesson M.I., Li X., Lee J.L., Ghosh S., Alsup J.W., Warner J.D., Tanaka M., Steward-Tharp S.M., Gadina M. (2011). Modulation of innate and adaptive immune responses by tofacitinib (CP-690,550). J. Immunol..

[31] Fernández-Clotet A., Castro-Poceiro J., Panés J. (2018). Tofacitinib for the treatment of ulcerative colitis. Exp. Rev. Clin. Immunol..

[32] Panés J., Vermeire S., Lindsay J.O., Sands B.E., Su C., Friedman G., Zhang H., Yarlas A., Bayliss M., Maher S. (2018). Tofacitinib in Patients with Ulcerative Colitis: Health-Related Quality of Life in Phase 3 Randomised Controlled Induction and Maintenance Studies. J. Crohns Colitis.

[33] Pantavou K., Yiallourou A.I., Piovani D., Evripidou D., Danese S., Peyrin-Biroulet L., Bonovas S., Nikolopoulos G.K. (2019). Efficacy and safety of biologic agents and tofacitinib in moderate-to-severe ulcerative colitis: A systematic overview of meta-analyses. United Eur. Gastroenterol. J..

[34] FDA Approves New Treatment for Moderately to Severely Active Ulcerative Colitis. https://www.fda.gov/news-events/press-announcements/fda-approves-new-treatment-moderately-severely-active-ulcerative-colitis.

[35] Evaluation of Oral Tofacitinib in Children Aged 2 to 17 Years Old Suffering From Moderate to Severe Ulcerative Colitis. https://clinicaltrials.gov/ct2/show/NCT04624230.

[36] Panés J., Sandborn W.J., Schreiber S., Sands B.E., Vermeire S., D’Haens G., Panaccione R., Higgins P.D.R., Colombel J.-F., Feagan B.G. (2017). Tofacitinib for induction and maintenance therapy of Crohn’s disease: Results of two phase IIb randomised placebo-controlled trials. Gut.

[37] Agrawal M., Kim E.S., Colombel J.-F. (2020). JAK Inhibitors Safety in Ulcerative Colitis: Practical Implications. J. Crohns Colitis.

[38] Dudek P., Fabisiak A., Zatorski H., Malecka-Wojciesko E., Talar-Wojnarowska R. (2021). Efficacy, Safety and Future Perspectives of JAK Inhibitors in the IBD Treatment. J. Clin. Med..

[39] Study to Evaluate the Efficacy and Safety of Filgotinib in the Induction and Maintenance of Remission in Adults with Moderately to Severely Active Ulcerative Colitis (SELECTION). https://clinicaltrials.gov/ct2/show/NCT02914522.

[40] https://www.ema.europa.eu/en/medicines/human/EPAR/jyseleca#assessment-history-section.

[41] Vermeire S., Schreiber S., Petryka R., Kuehbacher T., Hebuterne X., Roblin X., Klopocka M., Goldis A., Wisniewska-Jarosinska M., Baranovsky A. (2017). Clinical remission in patients with moderate-to-severe Crohn’s disease treated with filgotinib (the FITZROY study): Results from a phase 2, double-blind, randomised, placebo-controlled trial. Lancet.

[42] Troncone E., Marafini I., Del Vecchio Blanco G., Di Grazia A., Monteleone G. (2020). Novel Therapeutic Options for People with Ulcerative Colitis: An Update on Recent Developments with Janus Kinase (JAK) Inhibitors. Clin. Exp. Gastroenterol..

[43] Al-Bawardy B., Shivashankar R., Proctor D.D. (2021). Novel and Emerging Therapies for Inflammatory Bowel Disease. Front. Pharmacol..

[44] Mavropoulou E., Mechie N.-C., Knoop R., Petzold G., Ellenrieder V., Kunsch S., Pilavakis Y., Amanzada A. (2020). Association of serum interleukin-6 and soluble interleukin-2-receptor levels with disease activity status in patients with inflammatory bowel disease: A prospective observational study. PLoS ONE.

[45] Danese S., Vermeire S., Hellstern P., Panaccione R., Rogler G., Fraser G., Kohn A., Desreumaux P., Leong R.W., Comer G.M. (2019). Randomised trial and open-label extension study of an anti-interleukin-6 antibody in Crohn’s disease (ANDANTE I and II). Gut.

[46] Schreiber S., Aden K., Bernardes J.P., Conrad C., Tran F., Höper H., Volk V., Blase J.I., Nikolaus S., Bethge J. (2021). Therapeutic Interleukin-6 Trans-signaling Inhibition by Olamkicept (sgp130Fc) in Patients With Active Inflammatory Bowel Disease. Gastroenterology.

[47] Rothenberg M.E., Wang Y., Lekkerkerker A., Danilenko D.M., Maciuca R., Erickson R., Herman A., Stefanich E., Lu T.T. (2019). Randomized Phase I Healthy Volunteer Study of UTTR1147A (IL-22Fc): A Potential Therapy for Epithelial Injury. Clin. Pharmacol. Ther..

[48] Wagner F., Mansfield J., Geier C., Dash A., Wang Y., Li C., Lekkerkerker A., Lu T. (2020). P420 A randomised, observer-blinded phase Ib multiple, ascending dose study of UTTR1147A, an IL-22Fc fusion protein, in healthy volunteers and ulcerative colitis patients. J. Crohns Colitis.

